# Actions of Thyroid Hormone Analogues on Chemokines

**DOI:** 10.1155/2016/3147671

**Published:** 2016-07-17

**Authors:** Paul J. Davis, Gennadi V. Glinsky, Hung-Yun Lin, Shaker A. Mousa

**Affiliations:** ^1^Department of Medicine, Albany Medical College, Albany, NY 12208, USA; ^2^Pharmaceutical Research Institute, Albany College of Pharmacy and Health Sciences, Albany, NY 12144, USA; ^3^Institute of Engineering in Medicine, University of California, San Diego, CA 92093, USA; ^4^PhD Program for Cancer Biology and Drug Discovery, College of Medical Science and Technology, Taipei Medical University, Taipei 11031, Taiwan; ^5^Taipei Cancer Center, Taipei Medical University, Taipei 11031, Taiwan

## Abstract

The extracellular domain of plasma membrane integrin *α*v*β*3 contains a receptor for thyroid hormone (L-thyroxine, T_4_; 3,5,3′-triiodo-L-thyronine, T_3_); this receptor also binds tetraiodothyroacetic acid (tetrac), a derivative of T_4_. Tetrac inhibits the binding of T_4_ and T_3_ to the integrin. Fractalkine (CX3CL1) is a chemokine relevant to inflammatory processes in the CNS that are microglia-dependent but also important to normal brain development. Expression of the CX3CL1 gene is downregulated by tetrac, suggesting that T_4_ and T_3_ may stimulate fractalkine expression. Independently of its specific receptor (CX3CR1), fractalkine binds to *α*v*β*3 at a site proximal to the thyroid hormone-tetrac receptor and changes the physical state of the integrin. Tetrac also affects expression of the genes for other CNS-relevant chemokines, including CCL20, CCL26, CXCL2, CXCL3, and CXCL10. The chemokine products of these genes are important to vascularity of the brain, particularly of the choroid plexus, to inflammatory processes in the CNS and, in certain cases, to neuroprotection. Thyroid hormones are known to contribute to regulation of each of these CNS functions. We propose that actions of thyroid hormone and hormone analogues on chemokine gene expression contribute to regulation of inflammatory processes in brain and of brain blood vessel formation and maintenance.

## 1. Background

Appreciation of the existence of a plasma membrane receptor for thyroid hormone analogues on the extracellular domains of a structural plasma membrane protein, integrin *α*v*β*3 [1–3], has permitted recognition of new control mechanisms for the release of cytokines, including chemotactic cytokines or chemokines [4]. For example, transcription of the fractalkine ligand (CX3CL1) and receptor (CX3CR1) genes is downregulated in tumor cells by tetraiodothyroacetic acid (tetrac), a deaminated, naturally occurring analogue [5, 6] of L-thyroxine (T_4_), and this action is initiated at integrin *α*v*β*3 [2, 4]. These fractalkine effects were demonstrated in human breast cancer cells, and it is tumor cells and endothelial cells that generously express *α*v*β*3. Within the central nervous system (CNS), fractalkine has been observed to have both neuroprotective and neurotoxic actions [7] (see below) and we may ask if thyroid hormone in the CNS is one determinant of the type of action that CX3CL1 will manifest. Synthesis and release of a panel of cytokines is also regulated by thyroid hormone via *α*v*β*3 on the cell surface [8]. The integrin is generously expressed by nervous system tumor cells, for example, glioma [9] and glioblastoma cells [10], and proliferation of these cells is stimulated via *α*v*β*3 by L-thyroxine, the principal secretory product of the thyroid gland, and, to a lesser extent, by 3,5,3′-triiodo-L-thyronine (T_3_), which is derived from T_4_ by deiodination and is the form of thyroid hormone that is active intracellularly. Formulations of tetrac inhibit actions of T_4_ and T_3_ on nervous system cancer cells. It is important to note that healthy neurons also express *α*v*β*3 and that thyroid hormone has been shown to act on such cells to control functions such as sodium current (*I*_Na_) [11, 12]. Thus, binding to the plasma membrane of tumor cells, endothelial cells, and certain normal nervous system cells, thyroid hormone influences downstream the transcription of a number of genes relevant to inflammatory and immune responses [4, 8].

Thyroid hormone is widely acknowledged to have critical intracellular actions on the nervous system that are initiated by T_3_ at thyroid hormone receptors (TRs) in the cell nucleus. These hormonal effects that require primary interactions of T_3_ with TRs are described as genomic [13]. In contrast, nongenomic hormonal effects are those initiated at the plasma membrane or in cytoplasm [3, 13]; these may involve T_3_, T_4_, or hormone analogues such as tetrac. Actions initiated nongenomically may downstream involve gene transcription [3]. T_3_-dependent genomic effects are critical to central nervous development [14–16], neurophysiology [14], and neuroprotection [17–21]. It must also be pointed out that the hormone has actions on brain development that are nongenomic [22] and are chiefly upon the cytoskeleton and state of actin—soluble versus fibrous—within glial cells and neurons. These effects are critical to early structural development of brain, particularly the cerebellum, and are dependent upon T_4_, rather than T_3_.

In this position paper, we briefly review the actions of thyroid hormone analogues on chemokines that are relevant to the nervous system. Much of the information we have about these factors and their nongenomic regulation by thyroid hormone has come from studies of cells or tissues that are not of nervous system origin. However, studies of apparently neuroprotective properties of thyroid hormone have of course been conducted in nervous system tissues and are of particular interest because the hormone can be antiapoptotic and proangiogenic, qualities desirable in cells of the CNS in the setting of ischemia. But circumstances exist in which the hormone may also be proinflammatory and this quality may explain variability of reports in the thyroid hormone-neuroprotection literature. The current review is intended to encourage the examination in nerve cells and glia of the likely possibility that thyroid hormone analogues are modulators of chemokine actions in the nervous system.

## 2. Thyroid Hormone Analogues and Specific Chemokines

Human chemokines are 48 small (up to 14 kDa) chemotactic cytokines of four classes. The classes are CC, CXC, C, and CX_3_C, oriented about the conserved N-terminal cysteine, where X is any amino acid [25, 26]. Appending an L indicates the protein is a ligand and an R indicates function of the molecule as a receptor.

Chemokines may be constitutively expressed in tissues (“homeostatic” chemokines), for example, where they are involved in generation and maintenance of vasculature, or they may be periodically generated in response to local inflammatory responses. Both of these actions are observed in the nervous system. Thyroid hormone is an important proangiogenic factor by a variety of mechanisms [27, 28], contributing via this and other pathways to the inflammatory response [4], as pointed out above. It was thus not surprising to find that the thyroid hormone analogue, tetrac, reformulated as a nanoparticle (Nanotetrac or Nano-diamino-tetrac), affected transcription of the genes of as many as seven chemokines [4] with either of or both proangiogenic and proinflammatory properties (see below). The Nanotetrac formulation involves the covalent binding via a linker of tetrac to a large poly[lactic-co-glycolic acid] nanoparticle to maximize duration of exposure of tetrac to the cell exterior and integrin *α*v*β*3 [1]. These initial studies of thyroid hormone action on chemokine gene expression were conducted in tumor cells, but the nongenomic mechanism by which tetrac acts on chemokines involves plasma membrane integrin *α*v*β*3, a protein that mediates critical actions of thyroid hormone and hormone analogues on neurons [11, 12], granulocytes [29], and endothelial cells [1]. These cells are essential components of the homeostatic and inflammatory effects of chemokines in the nervous system and other tissues. The integrin also transduces the thyroid hormone (T_4_) proliferative signal on glioma [9] and glioblastoma [10] cells.

## 3. CC Chemokines and Thyroid Hormone Analogues

### 3.1. CCL20

Among the homeostatic chemokines in the CNS that contribute to maintenance of the vasculature of the blood-brain barrier is CCL20 [30]. CCL20 is designated a homeostatic chemokine, as defined by its constitutive expression in lymphatic and thymic tissue. It is also constitutively transcribed at low levels at the choroid plexus [31] and other epithelial barriers outside the nervous system, but its expression at these sites will respond to proinflammatory cytokines, for example, interleukin-6 (IL-6) [32]. CCL20 may play a role in the importation of T cells into the CNS [30, 31].

**Figure 1:**
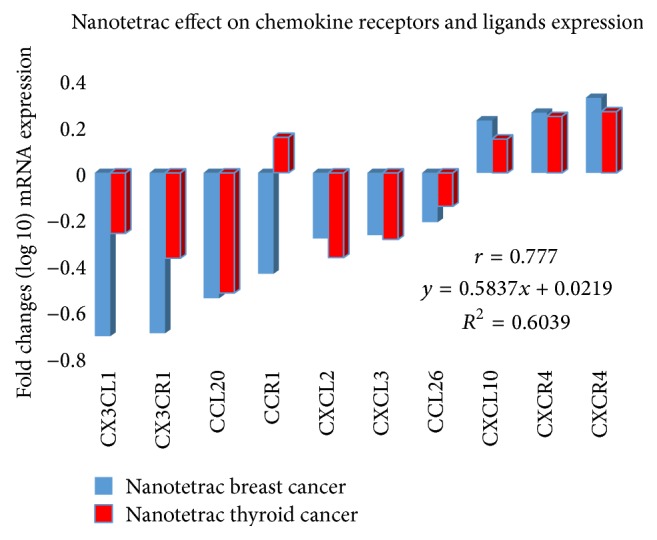
Highly correlated patterns of nanoparticulate tetrac (Nanotetrac) treatment of human breast and thyroid cancer cells on abundance of mRNAs of selected chemokine ligands and receptors. Shown are average values (log⁡10 transformed) of biological replicates of microarray analyses. Microarray gene expression methodology and data analysis pipelines are as previously reported [23, 24]. Use of two CXCR4 probes verified responsiveness to Nanotetrac of the intact gene and a variant of this chemokine. *r*, correlation coefficient; *R* ^2^, coefficient of determination used to estimate accuracy of the data model; *y*, results of linear regression analysis (regression line equation).

CCL20 gene transcription is downregulated by tetrac formulations (Figure 1) via an integrin *α*v*β*3-dependent process. Because T_4_ and T_3_ bind to the thyroid hormone-tetrac receptor on the extracellular domain of this integrin and this binding is inhibited by tetrac formulations, we propose that T_4_ and T_3_ have the capacity to stimulate CCL20 gene transcription. This possibility has not yet been investigated.

### 3.2. CCL26

CCL26 is another member of the CC family of chemokines whose gene transcription is regulated from the cell surface (integrin *α*v*β*3) by tetrac formulations. However, little is known about the actions of this chemokine on the CNS. Rather, CCL26 is involved in hepatoma cell biology and in pathogenesis of certain skin diseases [33].

## 4. CXC Chemokines and Thyroid Hormone Analogues

### 4.1. CXCL2

A product of microglia, chemokine CXCL2 has important chemotactic activity on granulocytes, inducing neutrophil infiltration of tissues with consequent inflammation and damage to tissues [34], as do other CXC products [35]. Release of CXCL2 from microglia is at least in part a response to increased tissue ATP levels that are a consequence of tissue damage. ATP activates signal transducing mitogen-activated protein kinase (MAPK) in these cells that downstream results in CXCL2 gene expression [34]. Traumatic brain injury-related inflammation involves choroid plexus production of CXCL2 and other CXCs that stimulate neutrophil infiltration [36]. CXCL2 is also implicated in the neutrophil infiltration of the brainstem in atypical experimental autoimmune encephalomyelitis (EAE) [37].

Decreased transcription of the CXCL2 gene is a tissue response to Nanotetrac (Figure 1), suggesting that this agent could have damage limitation activity in a variety of CNS tissue damage scenarios. The integrin-mediated effect of Nanotetrac also implies that agonist thyroid hormone (T_4_ or, possibly, T_3_) may act at *α*v*β*3 to enhance the inflammatory response to CNS tissue trauma or the process of EAE.

### 4.2. CXCL3

Like CXCL2, CXCL3 is also generated in the choroid plexus as a component of the inflammatory response to traumatic brain injury [36]. These chemokines are secreted by choroidal epithelium both basolaterally and apically, that is, bidirectionally, in order to assure granulocyte passage across the blood-brain barrier by the paracellular pathway.

In addition to its proinflammatory properties, CXCL3 has been shown to regulate the migration of cerebellar granule neuron precursor cells (GCPs) [36]. A significant minority of childhood medulloblastomas originate from GCPs, apparently reflecting defective migration of these cells and prolongation of their residence in the external granular layer (EGL) of the cerebellum. Such residence is associated with GCP proliferation and failure to differentiate. In this example of CXCL3 action, the potential contribution of thyroid hormone analogues is disparate and of particular interest. If, as we propose, T_4_ via its receptor on *α*v*β*3 enhances CXCL3 gene expression, then the presence of the hormone in the course of brain development would support the normal outmigration of GCPs from the EGL and contribute to minimization of risk of medulloblastoma. In contrast, the demonstrated action of tetrac in its nanoparticulate formulation to reduce CXCL3 gene expression might be a factor in decreased migration of GCPs; avoidance of exposure of the developing brain to tetrac is desirable.

### 4.3. CXCL10

The pathogenesis of certain autoimmune diseases of the CNS, such as multiple sclerosis (MS), remains incompletely understood. CXCL10 is a small proinflammatory, proangiogenic, interferon *γ*- (IFN-*γ*-) inducible chemokine that has been implicated in the development of MS [38]. CXCL10 is produced by white blood cells (granulocytes, monocytes), endothelial cells, and astrocytes, among others [38]. CXCL10 binds to the CXCR3 receptor that is expressed by T lymphocytes, natural killer (NK) cells, and certain kinds of epithelial cells. Vazirinejad and coworkers [38] and others [39–42] have found elevated circulating (serum) or CSF content of CXCL10 and have proposed that CXCL10 contributes importantly to inflammatory demyelination that is an essential component of MS. But CSF levels of CXCL10 are occasionally elevated in subjects with evidence of CNS inflammation [38, 43].

Because a tetrac formulation that acts exclusively at integrin *α*v*β*3* stimulates* transcription of the CXCL10 gene (Figure 1), the thyroid hormone-relevant issue that is raised here is whether thyroid hormone (T_4_ or T_3_)—through CXCL10—may be a factor that reduces the aggressiveness of pathogenesis of MS. Thus, it is important to examine the possible protective actions of T_4_/T_3_ in models of MS and a possibly deleterious effect of tetrac formulations in such models. That tetrac can downregulate expression of specific genes and upregulate other genes which is not surprising, given that thyroid hormone analogues via *α*v*β*3 can, via signal transduction pathways, differentially control proactivator and corepressor nucleoproteins. We have reported elsewhere that tetrac upregulates expression of thrombospondin 1 (*TSP1*) gene and microRNA-15A (miR-15A) but downregulates miR-21,* EGFR*, and* VEGFA* [2].

Against this background, it is important to note that thyroid hormone has been shown by others to induce remyelination [44] via action(s) on oligodendrocyte precursor cells in the model of cuprizone-induced demyelination. Dell'Acqua and coworkers [18] also have shown that the hormone also supports remyelination and is neuroprotective in EAE.

Thus, the thyroid hormone analogue, tetrac (formulated as Nano-diamino-tetrac), has the potential to modulate CNS inflammation bidirectionally by action on expression of the genes for CXCL2 and CXCL3—supporting inflammation—and on expression of the CXCL10 gene, possibly reducing inflammation. Testing in models of CNS inflammation will determine whether there is a dominant thyroid hormone/tetrac effect on one or more specific CXC chemokines.

## 5. C Chemokines

The C chemokines are XCL1 (lymphotactin-*α*) and XCL2 (lymphotactin-*β*). The single receptor to which these chemokines bind is XCR1. The XCL1, XCL2, and XCR1 genes are apparently not subject to modulation by thyroid hormone or the hormone analogue, tetrac.

## 6. CX3C Chemokines and Thyroid Hormone Analogues

### 6.1. CX3CL1

The resident macrophages of the CNS, microglia, have both proinflammatory (M1) and anti-inflammatory (M2) phenotypes [45]. Release of CX3CL1 (fractalkine) by damaged neurons supports an M1 response by binding to its receptor (CX3CR1) on microglia and fostering recruitment of circulating white blood cells. The proinflammatory state ensues with release of factors such as reactive oxygen species, nitric oxide, and inflammatory cytokines. The M2 state is the product of microglial production of anti-inflammatory cytokines and growth factors in response to CX3CL1 at the microglial CX3CR1 [5, 45]. Thus, the M1 versus M2 phenotypic state of microglia [46, 47] appears to determine neuroprotective versus neurotoxic activities of CX3CL1 [48] and the evolution/progression of brain diseases such as Alzheimer's [49], ischemia, and traumatic brain injury [7, 46–48].

Fractalkine and its receptor, CXCR1, may form a ternary complex with integrin *α*v*β*3 that results in activation of the integrin [50]; activation is a change in configuration of the protein to support cell migration and cell-cell interactions that are critical functions of integrins. Microglia express *α*v*β*3 [51]. Inhibiting thyroid hormone actions at integrin *α*v*β*3, Nanotetrac decreases transcription of the CX3CL1 gene [4] and thus we speculate that the M2 and M1 responses of microglia are both supported by nongenomic action of T_4_ at *α*v*β*3. This possibility has not been examined experimentally, nor has the possibility been considered that the transition from M2 to M1 state in microglia is a process that is subject to influence by thyroid hormone.

Thyroid hormone in the form of T_3_ is known to have* genomic* effects on microglia [52, 53]. The nongenomic downregulation of CX3CL1 gene expression by Nanotetrac in damaged neurons is possibly a desirable intervention to investigate in the early phases of models of Alzheimer's. In contrast, such an intervention is to be avoided in settings in which the M2 response prevails, for example, in recruitment of microglia to developing synapses in developing brain [54] and regulation of microglia-neuron interactions in brain development, adulthood, and aging [55]. Because thyroid hormone can provoke inflammatory cytokine production [4], one can ask whether this hormone permissively contributes to induction of the M1 response, in which hormonal action on fractalkine production could be either protective or neurotoxic. Like Lauro and collaborators [7], we endorse additional studies of the determinants of the neuroprotective versus neurotoxic CX3CL1 responses; we also urge the definition of the possibly distinctive roles of thyroid hormone isoforms in the M1 and M2 responses.

The actions of fractalkine on the developing brain have been recently reviewed by Arnoux and Audinat [54]. The functions in developing brain of CX3CL1 on microglia—through CX3CR1 on glial cells—include support of neuron survival and axon outgrowth and refining synaptic circuits through microglial phagocytic activity. There is also some neuronal death that occurs normally in brain development and microglia are involved in such events. Because expression of the CX3CL1 gene is downregulated by the thyroid hormone analogue, tetrac, at integrin *α*v*β*3, we propose that the critical relevance of T_3_ and T_4_ to normal brain development is in part dependent upon actions of the hormone on the fractalkine gene. Tetrac has anticancer properties outside the CNS and any clinical use of the agent or its formulations that may emerge must be avoided in the setting of pregnancy.

Finally, it has recently been appreciated that fractalkine can bind directly to integrin* independently* of CX3CR1 [56] and thereby activate the integrin. The binding site where this occurs is near the RGD (Arg-Gly-Asp) recognition site in the head of the integrin and thus proximal to the thyroid hormone-tetrac receptor on *α*v*β*3. Thus, CX3CL1 may have integrin-dependent functions in cells that do not express CX3CRI. The existence of this chemokine binding site on the integrin also raises the possibility of interactions/crosstalk between the hormone-binding and fractalkine-binding sites on the integrin that could be influenced by tetrac and T_4_. CX3CL1 is the only chemokine known to undergo constitutive cell internalization [57] and thyroid hormone is known to drive internalization of *α*v*β*3 [58]. The possibility thus exists that cell uptake of fractalkine is modulated by thyroid hormone binding to the integrin.

## 7. Chemokine Receptor Genes

The chemokine receptor genes whose transcription is subject to modulation by thyroid hormone analogue tetrac include (1) CXCR4, the principal ligand of which is CXCL12 [59] and the transcription of the gene which is increased by Nanotetrac; (2) CCR1, the ligands which include CCL3, CCL4, CCL6, CCl9/CCL10, CCL14, CCL15, and CCL23 [59], and the transcription of the gene which is decreased by Nanotetrac; and (3) CX3CR4, the ligand of which is CX3CL1. Transcription of this receptor gene is frankly decreased by Nanotetrac. Only in the case of CX3CR4/CX3CL1 are both ligand and receptor genes affected similarly from the thyroid hormone-tetrac receptor on *α*v*β*3. Receptor gene responses to Nanotetrac are shown in Figure 1.

We propose that agonist thyroid hormone, for example, T_4_, acts contrarily to Nanotetrac at the integrin—this would involve a decrease in CXCR4 gene expression and increases in CCR1 and CX3CR4 gene transcription—but this has not been experimentally approached.

## 8. Discussion

Among the principal issues of this review is the relevance of integrin *α*v*β*3 to regulation of chemokine gene expression. The integrin has been seen primarily to bind key extracellular matrix proteins—fibronectin, vitronectin, and osteopontin [60], as examples—that are critical to tissue integrity, and only recently has it been recognized that small molecule ligands of the integrin of the thyroid hormone family specifically affect transcription of at least 6 chemokines. Of these agents, 5 are important to functions of the CNS, particularly, maintenance of the integrity of the choroid plexus and blood-brain barrier, and contributions to inflammatory processes in the nervous system.

The fact that thyroid hormone analogues can affect chemokine ligand and receptor gene transcription is not surprising, given the actions of analogues of the hormone on several aspects of the inflammatory response [4] and on the immune response [8]. We have previously pointed out that expression of the genes for CX3CL1 (fractalkine) and the fractalkine receptor is subject to regulation from the thyroid hormone-tetrac receptor on integrin *α*v*β*3 [4]. We emphasize here that observations of effects of nanoparticulate tetrac (Nanotetrac) on expression of chemokine genes do not provide assurance that principal thyroid hormone isoforms—T_4_ and T_3_—affect expression of all of these genes and do so in directions opposite to those of Nanotetrac. That this may be the case, however, is suggested in the case of regulation of demyelination/remyelination in several models. That is, thyroid hormone has remyelination activity [42], whereas the antithyroid hormone factor, tetrac, has been shown by us to* stimulate* CXCL10 expression, supporting demyelination. It is thus important to address this issue directly and compare integrin-mediated studies of T_4_/T_3_ versus tetrac on transcription of CXCL10 and other chemokine genes, particularly in cells of importance to vascular and inflammatory responses of the CNS.

In contrast to its upregulatory action on CXCL10 gene expression, tetrac in the case of other chemokines reviewed here serves as a factor downregulating gene expression. We know that T_4_ supports the inflammatory response in a variety of tissue settings [4], and therefore the suppressive effects of tetrac on expression of genes for CXCL2, CXCL3, and CCL20 would be anti-inflammatory.

T_4_ and unmodified or reformulated tetrac could affect the state of inflammatory cells outside the CNS, following which these cells could gain access to the nervous system. However, T_4_ and tetrac are avidly bound at a receptor site on plasma protein transthyretin (TTR), and TTR serves at the choroid plexus to support trafficking across the blood-brain barrier of thyroid hormone analogues [61]. Thus, two thyroid hormone receptors are of particular interest to the actions of thyroid hormone and hormone analogues on specific chemokine gene expression in the CNS: TTR and integrin *α*v*β*3. These receptors are structurally unrelated, but essential to provision of access of hormone analogues to various cells in the brain, for example, microglia, astrocytes, neurons, and endothelial cells, that are targets of specific chemokines.

In summary, thyroid hormone is proposed to support proinflammatory processes in the CNS that are due to a limited number of chemokines. Thyroid hormone, especially T_4_, is proangiogenic by a number of mechanisms in a variety of tissues [28] and thus it is not surprising that thyroid hormone analogues affect angiogenesis in brain, at least in part via chemokines. Thyroid hormone is a proliferative factor for gliomas [9] and glioblastoma [10], as well as for a variety of other nonneurological cancers [1, 3, 62]. It is possible that the biology of another CNS tumor, medulloblastoma, may be affected by thyroid hormone and hormone analogues, but in a wholly different manner and working via a chemokine, CXCL3 [36]. That is, via stimulation of CXCL3 gene transcription, thyroid hormone might increase outmigration of cerebellar granule neuron precursor cells from the external granular layer of the cerebellum and* reduce* the risk of medulloblastoma in pediatric patients. Tumor cells alter the microenvironment, in part, by releasing chemokines. Consistent interference with transcription of the chemokine genes that we observed in the two examples of human cancer (Figure 1) may constitute an important and yet underappreciated molecular component of the mechanisms of anticancer activity of therapeutic tetrac formulations.
